# Biomarkers of Metabolism and Inflammation in Individuals with Obesity and Normal Weight: A Comparative Analysis Exploring Sex Differences

**DOI:** 10.3390/ijms26157576

**Published:** 2025-08-05

**Authors:** Eveline Gart, Jessica Snabel, Jelle C. B. C. de Jong, Lars Verschuren, Anita M. van den Hoek, Martine C. Morrison, Robert Kleemann

**Affiliations:** 1 Department of Metabolic Health Research, The Netherlands Organisation for Applied Scientific Research (TNO), 2333 BE Leiden, The Netherlands; 2 Department of Microbiology and Systems Biology, The Netherlands Organisation for Applied Scientific Research (TNO), 2333 BE Leiden, The Netherlands

**Keywords:** metabolic risk factors, immunoassays, obesity, sex differences

## Abstract

Blood-based biomarkers allow monitoring of an individual’s health status and provide insights into metabolic and inflammatory processes in conditions like obesity, cardiovascular, and liver diseases. However, selecting suitable biomarkers and optimizing analytical assays presents challenges, is time-consuming and laborious. Moreover, knowledge of potential sex differences remains incomplete as research is often carried out in men. This study aims at enabling researchers to make informed choices on the type of biomarkers, analytical assays, and dilutions being used. More specifically, we analyzed plasma concentrations of >90 biomarkers using commonly available ELISA or electrochemiluminescence-based multiplex methods, comparing normal weight (BMI < 25; *n* = 40) with obese (BMI > 30; *n* = 40) adult blood donors of comparable age. To help choose optimal biomarker sets, we grouped frequently employed biomarkers into biological categories (e.g., adipokines, acute-phase proteins, complement factors, cytokines, myokines, iron metabolism, vascular inflammation), first comparing normal-weight with obese persons, and thereafter exploratively comparing women and men within each BMI group. Many biomarkers linked to chronic inflammation and dysmetabolism were elevated in persons with obesity, including several adipokines, interleukins, chemokines, acute-phase proteins, complement factors, and oxidized LDL. Further exploration suggests sex disparities in biomarker levels within both normal-weight and obese groups. This comprehensive dataset of biomarkers across diverse biological domains constitutes a reference resource that may provide valuable guidance for researchers in selecting appropriate biomarkers and analytical assays for own studies. Moreover, the dataset highlights the importance of taking possible sex differences into account.

## 1. Introduction

Blood-based biomarkers are frequently used in biomedical research, for instance to characterize the health status of subjects or to monitor their response to a treatment. These biomarkers are unique compared to other health readouts, such as body weight or blood pressure, because blood-based biomarkers can provide insight into underlying metabolic or inflammatory aberrations that drive the development of diseases [1]. Although biomarkers are an important tool in biomedical research, it is difficult and time-consuming to define the most optimal biomarker set, identify robust assays and define optimal dilutions to reduce matrix effects from serum or plasma constituents. Furthermore, little is known about potential sex differences regarding biomarker expression and concentrations in the circulation. For biomarkers associated with inflammatory and coagulation processes (e.g., cytokines, chemokines, complement factors), plasma is typically used or should be used (e.g., fibrinogen, plasminogen activator inhibitor (PAI-1)) [2,3,4]. In contrast to plasma, during the preparation of serum, blood-clotting activates platelets which affects biomarker concentrations because platelets themselves can release, among others, cytokines, chemokines, and coagulation factors as well as extracellular vesicles (direct effects), or affect immune cells (via indirect effects) [5,6].

A reason for of the limited knowledge about sex differences is that women are often under-represented in biomedical research [7]. Women of childbearing age have been excluded from clinical trials mainly due to safety reasons, and for safety of their (unborn) offspring [8]. Moreover, hormone levels vary throughout a woman’s life (e.g., monthly cycles, puberty, pregnancy and menopause) which often results in more variable readouts, as sex hormones, particularly estrogen, influence disease development strongly [9]. Lastly, women are often the caregivers in their family [10], which compromises their participation in trials because of time and logistic constraints. As a consequence, a considerable portion of our current knowledge about biomarkers that are used during diagnosis, treatment, and prevention of disease originates from studies performed exclusively in men or over-represented with men [8,11]. Better insight into possible differences in biomarker levels between women and men is thus important for improving diagnostic accuracy, gaining insight into the pathogenesis of diseases, and interpreting treatment effects.

Obesity is the most prevalent metabolic disease, associated with many comorbidities including type 2 diabetes (T2D), metabolic dysfunction-associated steatotic liver disease (MASLD), certain cancers, and cardiovascular disease (CVD) [12]. Obesity-associated diseases are characterized by metabolic abnormalities and often a state of chronic inflammation both of which are reflected in the circulation [12]. It is well-established that obesity is more prevalent among women than men [13], but little is known about possible differences in circulating biomarkers between the sexes.

Biomarkers are broadly applied in biomedical research, and they are analyzed in both healthy subjects and those who are at risk of, or suffer from, diseases. In the case of obesity and development of cardiometabolic comorbidities, typical biomarkers informing about the metabolic health of a person include endocrine factors and modified lipoprotein particles (e.g., oxidized low-density lipoprotein (LDL)). In addition, biomarkers that inform on inflammatory processes are of particular interest because chronic inflammation is a critical driver of the aforementioned comorbidities. Among these are mediators of the complement system (complement factors C1q, C3, C5), cytokines (e.g., interleukins, tumour necrosis factor alpha (TNF-a)), as well as acute-phase response proteins such as C-reactive protein (CRP) or serum amyloid A (SAA) [14]. Biomarkers of vascular inflammation include selectins (e.g., E-selectin) and vascular adhesion molecules like soluble vascular cell adhesion molecule (sVCAM)-1 or soluble intracellular adhesion molecule (sICAM)-1 that can be shed from endothelial cells and subsequently measured in the circulation [15].

In this explorative and inventory study we used standardized well-established Enzyme-Linked Immunosorbent Assay (ELISA) and multiplex immunoassays to analyze the plasma concentrations of a broad number of biomarkers in normal-weight and obese women and men. The biomarkers were grouped into different biological categories (e.g., adipokines, acute-phase proteins, cytokines, etc.) to illustrate their associations with organs and disease processes. We first compared biomarker concentrations in a normal-weight group (BMI < 25) versus the obese group (BMI > 30) independent of sex and, subsequently, we explored differences between women and men within each BMI group. This explorative analysis resulted in a comprehensive biomarker dataset with biomarker concentrations that can be used to guide biomarker approaches and that may serve as a reference dataset, indicating the existence of possible sex differences in many biomarker categories. The methods used are described in such a way (detailing assays used, suppliers, and dilutions of plasma) that they can easily be adopted by other researchers enabling accurate biomarker analyses and a more nuanced interpretation of results.

## 2. Results

**Figure 1:**
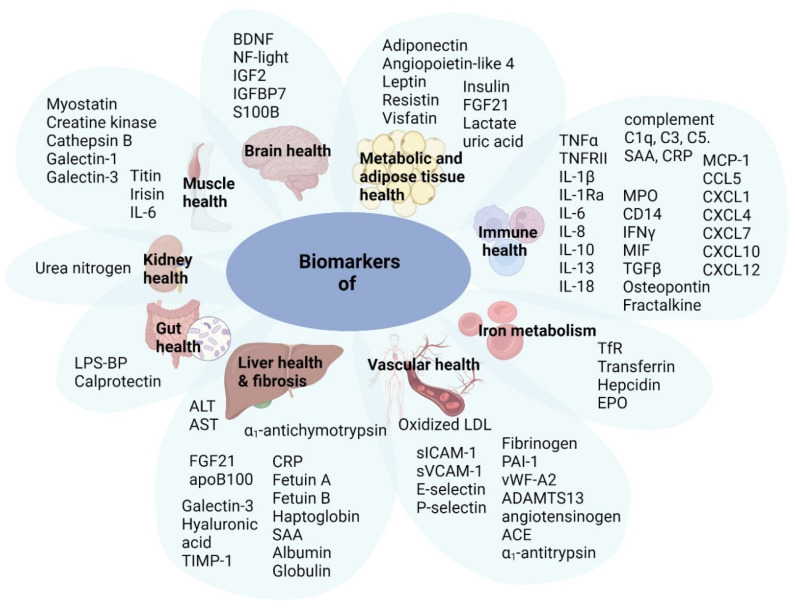
Overview of analyzed biomarkers. Biomarkers were clustered based on the organ or the biological process they are typically associated with in the literature. Notably, most of these biomarkers are not very organ-specific and may thus be expressed by several organs at the same time, at different rates. Biomarkers that are ubiquitously expressed or that cannot be linked to a particular process were omitted. Abbreviations (in alphabetical order): ACE, angiotensin converting enzyme; ADAMTS13, a disintegrin and metalloproteinase with thrombospondin motifs 13; ALT, alanine aminotransferase; AST, aspartate aminotransferase; BDNF, brain-derived neurotrophic factor; CCL5, C-C motif chemokine ligand 5 (also referred to as RANTES); CRP, C-reactive protein; CD14, cluster of differentiation 14; CXCL, C-X-C motif chemokine ligand; EPO, erythropoietin; FGF21, fibroblast growth factor 21; IFN-γ, interferon-gamma; IL, interleukin; IL-1Ra, interleukin-1 receptor antagonist; IGF2, insulin growth factor 2; IGFBP7, insulin growth factor binding protein 7; LPS-BP, LPS binding protein; MCP-1, monocyte chemoattractant protein-1; MIF, macrophage migration inhibitory factor; MPO, myeloperoxidase; NF-light, neurofilament light chain; PAI-1, plasminogen activator inhibitor-1; S100B, S100 calcium-binding protein B; SAA, serum amyloid A; TfR, transferrin receptor; sICAM-1, soluble intracellular cell adhesion molecule-1; TIMP-1, tissue inhibitor of metalloproteinases-1; sVCAM-1, soluble vascular cell adhesion molecule-1; TGF-beta, transforming growth factor-beta; TNF-α, tumour necrosis factor-alpha; TNFRII, tumour necrosis factor receptor II; vWF, van Willebrand factor.

The biomarkers measured in this study were grouped into categories based on their associations with a biological process or the health status of an organ (Figure 1).

### 2.1. Obesity-Associated Differences in Circulating Biomarkers Independent of Sex

**Figure 2:**
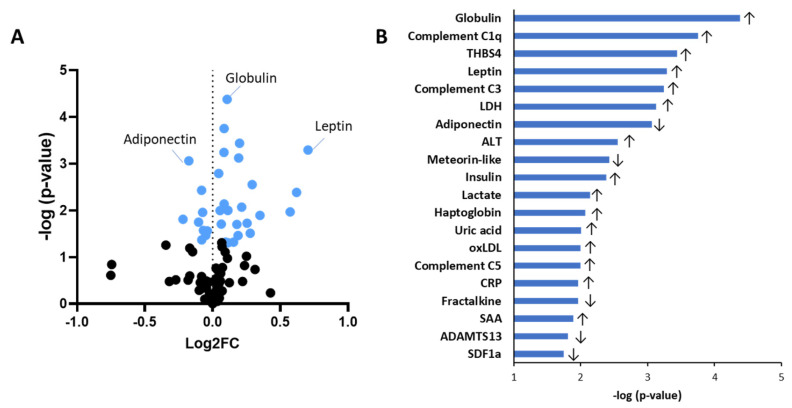
Obesity-associated differences in circulating biomarkers independent of sex. Volcano plot of biomarkers in obese vs. normal-weight persons. (**A**) Positive (or negative) log fold change (Log2FC) means that the respective biomarker is increased (or decreased). Blue dots refer to biomarkers that were significantly differentially expressed in all obese vs. all normal-weight persons, as illustrated by adiponectin, globulin, and leptin. Black dots refer to biomarkers that did not significantly differ comparing all obese vs. all normal-weight persons. (**B**) List of the top 20 significant biomarkers (−log *p*-value), the arrows indicate whether a particular biomarker is increased or decreased in obese compared to normal-weight persons. Abbreviations (in alphabetical order): ADAMTS13 = a disintegrin and metalloproteinase with thrombospondin motifs 13; ALT = alanine transaminase; CRP = C-reactive protein; LDH = lactate dehydrogenase; oxLDL = oxidized low-density lipoprotein; SAA = serum amyloid A; SDF-1a = stromal cell-derived factor 1 alpha; THSB4 = thrombospondin-4.

We first compared biomarker plasma concentrations between the obese and normal-weight groups independent of sex (Figure 2; Table 1). A volcano plot analysis showed that in total 56 biomarkers were numerically higher in the obese compared to the normal-weight individuals, while 39 biomarkers were lower in the obese (Figure 2A). The most significantly different biomarkers are listed in Figure 2B and include biomarkers of inflammation such as globulin and C-reactive protein (CRP), complement factors C1q and C3, as well as metabolic biomarkers such as leptin and adiponectin. The following differences were observed for the various biomarker categories: As expected, in the obese group, the adipokine adiponectin was significantly reduced relative to normal-weight individuals, whereas leptin was significantly increased (Table 1). No significant differences between obese and normal-weight groups were found for angiopoietin-like 4, resistin or visfatin.

Proteins involved in the onset and amplification of inflammation were significantly increased in the obese group and included cluster of differentiation (CD)-14, interleukin (IL)-6, IL-18 and tumour necrosis factor-receptor II (TNF-RII). No significant differences were found for calprotectin S100A8, calprotectin S100A8/S100A9, interferon (IFN)-γ, IL-1b, IL-1Ra, IL-8, IL-10, IL-13, macrophage migration inhibitory factor (MIF), myeloperoxidase (MPO), transforming growth factor (TGF)-β, C-C motif chemokine ligand (CCL)-5, C-X-C motif chemokine ligand (CXCL)-4, CXCL-7, CXCL-10 and osteopontin. Moreover, in the obese group inflammation-associated biomarkers belonging to the complement system were significantly increased, including complement C1q, C3 and C5. Also, several positive acute-phase response proteins were elevated in the obese group: CRP, haptoglobin and serum amyloid A (SAA). No significant changes were observed for LPS-binding protein (LPS-BP) and α1-antichymotrypsin as well as for the negative acute-phase response proteins fetuin-A and fetuin-B.

Furthermore, the obese participants showed a significant increase in an oxidative stress-associated biomarker, oxidized low-density lipoprotein (ox-LDL) particles.

The liver integrity marker aspartate transaminase (AST) was unaffected, whereas alanine transaminase (ALT) was significantly increased in the obese group. Circulating biomarkers that are associated with liver fibrosis hyaluronic acid was significantly elevated in the obese group, while galectin-3 and tissue inhibitor of metalloproteinase (TIMP)-1 were comparable in both groups.

Plasma concentrations of the transferrin receptor (TfR) implicated in iron transport across membranes, was significantly elevated in the obese group, while the major iron-carrier protein in plasma, transferrin, was lower. No differences between the groups were found for hepcidin or erythropoietin (EPO), both of which are also involved in iron metabolism.

Biomarkers associated with coagulation, thrombosis, and fibrinolysis such as alpha-1 antitrypsin (A1AT), fibrinogen, and plasminogen activator inhibitor (PAI)-1 were comparable between obese and normal-weight groups. Similarly, there was no difference between the obese and normal-weight groups for the blood pressure modulators angiotensinogen and angiotensin converting enzyme (ACE).

Neurological factors brain-derived neurotrophic factor (BDNF), neurofilament (NF)-light, insulin growth factor (IGF)-2, insulin growth factor-binding protein (IGFBP)-7 and S100 calcium-binding protein B (S100B) levels were comparable in both groups.

Biomarkers associated with muscle health including creatine kinase and titin were found to be significantly elevated in the obese group, whereas myostatin, cathepsin B, galectin-1, and irisin were comparable in both groups.

Kidney health factor urea nitrogen was comparable in both groups.

Lastly, other biomarkers of which the function to a specific organ or biological system is less established such as lactate dehydrogenase (LDH), uric acid, thrombospondin (THBS)-4, and meteorin-like were found to be increased in obese humans. Obese and normal-weight groups were comparable with respect to cystatin C and peptidyl glycine alpha-amidating monooxygenase (PAM).

### 2.2. Sex Differences in Biomarker Expression in the Lean Group

To study putative sex differences, we first compared biomarker concentrations between normal-weight women and men (Table 1). The data indicated that the adipokine leptin was higher in normal-weight women than in normal-weight men. In the category ‘onset and amplification of inflammation’ the biomarkers CCL2, osteopontin and complement C5 were significantly lower in normal-weight women than in normal-weight men. Furthermore, women had lower concentrations of soluble vascular cell adhesion molecule (sVCAM)-1, a biomarker that is expressed by endothelial cells. Lastly, the muscle health biomarker creatine kinase and kidney health biomarker urea nitrogen were found to be lower in normal-weight women. No further significant differences were found between normal-weight women and men.

### 2.3. Sex Differences in Biomarker Expression in the Obese Group

We next examined possible sex differences in the context of obesity and compared obese women with obese men (Table 1).

The concentrations of the adipokine leptin and the cytokine tumour necrosis factor (TNF)-α were found to be higher in obese women compared to obese men. A larger number of biomarkers were, however, significantly lower in obese women than in obese men: these biomarkers were not limited to a particular category and included inflammatory cytokine MIF, vascular inflammation marker E-selectin, positive acute-phase response protein LPS-BP, metalloproteinase inhibitor TIMP1, serine proteinase inhibitor PAI-1, and urea nitrogen, all of which were significantly lower in obese women. The concentrations of the other biomarkers were statistically comparable between obese women and men.

Altogether, these comparisons showed so far that a substantial number of biomarkers are differentially expressed in women when compared to men. These differences were observed in the lean condition and the obese condition. The former indicates that the set point of a biomarker may differ between sexes, while the latter indicates that the magnitude by which a biomarker changes during obesity development may also differ between men and women.

### 2.4. Sex-Specific Biomarker Direction Regulation

**Figure 3:**
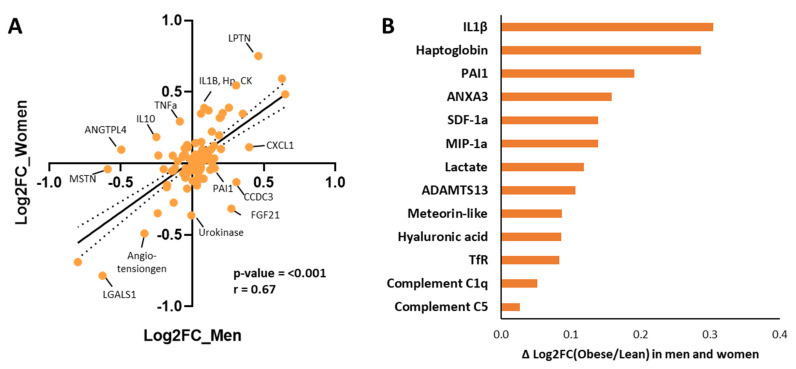
Sex-specific differences in biomarker regulation visualized in a correlation plot. (**A**) Sex-specific changes in biomarker levels in which the *y*-axis is the log fold change in a biomarker in obese women vs. normal-weight women (Log2FC_women). Similarly, the *x*-axis represents the log fold change in a biomarker in obese men vs. normal-weight men (Log2FC_men). Biomarkers similar in women and men fall directly onto the linear regression line, whereas biomarkers with an offset from the linear regression line behave differently in women and men, suggesting dissimilar regulation in the two sexes. A selection of biomarkers is indicated, along with their names. (**B**) The top 13 significant biomarkers were sorted on strongest absolute difference between obese and normal-weight individuals in men and women as delta log fold change on the *x*-axis. To generate the graphs, biomarker expression values were plotted, and Pearson regression analysis was performed to obtain the correlation coefficient r. Abbreviations of biomarkers (in alphabetical order): ADAMTS13 = a disintegrin and metalloproteinase with thrombospondin motifs 13; ANGTPL4 = angiopoietin like-4; CK = creatine kinase; Hp = haptoglobin; IL-1 beta = interleukin-1beta; LGALS1 = galectin 1; LPTN = leptin; MIP-1alpha = macrophage inflammatory protein-1 alpha (or CCL3); MSTN = myostatin; PAI-1 = plasminogen activator inhibitor-1; SDF-1a = stromal cell-derived factor 1 alpha; TfR = transferrin receptor.

Lastly, we studied whether the magnitude and direction of biomarker changes are similar in both sexes between the obese and normal-weight condition to gain insight into possible dissimilar regulations in the sexes. This was achieved by comparing the direction of the change from the normal weight to the obese condition in women and in men (Figure 3). The biomarker changes are visualized in a correlation plot (Figure 3A), which revealed a strong overall correlation (*p* < 0.001, R = 0.67). More specifically, biomarkers that are plotted directly onto the linear regression line appear to have a highly comparable direction of change in women and men suggesting similar (transcriptional and translational) regulation in both sexes, By contrast, the biomarkers with offset from the linear regression line appear to have sex-specific differences changes which suggests sex-specific differences in biomarker regulation or biomarker stability. The 13 biomarkers with greatest offset from the regression line are highlighted in Figure 3B, sorted based on the absolute difference in their change in women and men. For example, IL-1β and haptoglobin (Hp) in the upper right quadrant are both increased in men and women from the normal weight to the obese condition; however, this change is much greater in women than men. Another example, PAI-1 located in the bottom right quadrant, is a biomarker which increased in obese men, while it slightly decreased in obese women. Performing statistical comparisons on the subgroups, obese versus normal-weight women and obese vs. normal-weight men, we found that the following biomarkers were significantly different in both men and women: THBS4, IL6, leptin, globulin, and LDH.

## 3. Discussion

This study is a comprehensive analysis of biomarkers that are frequently employed in biomedical research to characterize health states of persons and the development of metabolic-inflammatory disorders. The study resulted in (i) an overview of biomarkers that were clustered based on their associations with organs and biological processes and (ii) a large biomarker dataset with plasma biomarker concentrations of obese and normal-weight individuals and respective methodological information. Altogether, this reference resource may provide guidance in defining adequate sets of biomarkers and assays, and the provided biomarker expression data may help other investigators make informed choices on the required dilutions, saving time and resources during biomarker analyses. The biomarkers (Table 1) (were clustered in biological categories (e.g., adipokines, cytokines, vascular inflammation markers, iron metabolism markers, etc.) to gain insight into metabolic or inflammatory derangements related to obesity. A subsequent exploration of putative sex differences revealed some important sex differences in both the normal-weight group and the obese group. Raising awareness about the existence of putative sex differences in biomarker expression may help other researchers to optimize their design of studies, data analysis and interpretation of biomarker results. Of note, biomarker analyses were performed in plasma (and not serum) because we analyzed biomarkers implicated in coagulation (e.g., fibrinogen, PAI-1, various complement factors), and because the coagulation process can activate platelets which can affect biomarker levels directly (e.g., release of chemokines) or indirectly [5,6].

In this study, the obese phenotype was associated with significant changes in cardiometabolic risk factors, including several plasma markers of dysmetabolism and inflammation. More specifically, we found that the obese group had lower plasma concentrations of adiponectin and higher levels of leptin. The observed increase in metabolic inflammatory markers is in line with previous studies in obese subjects, as these markers are known to correlate with fat mass [16]. Conversely, loss of fat mass, for instance after bariatric surgery, impacts adipokines resulting in an increase in adiponectin and decrease in leptin [2] or acute-phase proteins like CRP or SAA. Consistent with this, the acute-phase reactants CRP, haptoglobin and SAA were found to be higher in the obese subjects compared to normal-weight individuals. These acute-phase proteins are secreted by the liver and regulated by cytokines, including IL-6 and TNF-α, which are linked to morphological changes in adipose tissue [3] and may be released from visceral adipose tissue depots [16]. In the obese group, several inflammatory cytokines were increased in plasma such as IL-6, IL-18 and the cytokine receptor TNF-RII, as well as complement system factors (C1q, C3 and C5). The obese inflammatory phenotype was also associated with increases in plasma ox-LDL. The LDL particle in particular is sensitive to oxidative stress, and several of its constituents can become oxidized. This results in a modified particle referred to as ox-LDL which is implicated in the process of atherogenesis and the development of CVD by activating endothelial cells and thereby increasing immune cell infiltration [17].

In obese subjects, circulating levels of transferrin receptor (TfR) were increased. TfR is a membrane receptor involved in the control of iron supply to the cell through the binding of transferrin. Transferrin is the major iron-carrier protein in the circulation [18], and the concentration of this protein was found to be reduced in the obese group. A potential connection between iron trafficking and obesity is that cytokines can alter iron trafficking, allowing monocytes to take up iron via the TfR-mediated pathway [19]. An increased iron uptake by monocytes under chronic inflammatory conditions can thus contribute to iron deficiency in obese individuals [20].

Other biomarkers that have previously been linked to obesity were also found to be increased, including THBS4, which mediates cell communication and meteorin-like [21]. This is thought to be an adaptive response and protective because of the beneficial metabolic (i.e., glucose homeostasis) and anti-inflammatory properties of meteorin-like [22].

There is increasing interest in understanding the sex differences underlying disease risk (e.g., for CVD) and, for instance, young adult overweight men have a higher mortality risk than women [23,24]. It is, however, less clear whether such sex differences in disease risk translate into differences in biomarkers, although studies with gender-affirming hormone therapy do suggest so [4]. Better insight into possible differences in biomarker expression between women and men can be important for the understanding of disease risk, but also for diagnosis and treatment. A limitation of the present study is that male/female ratio was not balanced, and that the group sizes were relatively small, which is why we performed the analysis of putative sex differences as an explorative analysis. Hence, the small sample size limits the statistical power and the possibility to draw firm conclusions. Other limitations are related to our choice to use plasma from blood donors which is commercially available in large quantities (which were needed to test and set up each biomarker assay in a series of piloting experiments). As a consequence of using anonymized donor blood (and thus an observational design), we had no data available on fat distribution, menstrual cycle phase, or possible undiagnosed metabolic diseases, nor did we have longitudinal blood samples. For future biomarker studies, we recommend using samples from preferably longitudinal clinical studies with more detailed information on the participants. In the present study, we observed that some biomarkers seem to differ between the sexes in the normal-weight situation: the adipokine leptin was higher in women compared to men. Leptin is a hormone predominantly secreted by adipocytes from subcutaneous fat, which is the dominant form of fat storage in women and thus may contribute to higher leptin levels than those observed in men [25]. However, leptin may also be regulated by sex steroids, insulin, glucocorticoids, and cytokines [26]. The latter seems to be less likely to play a large role in the current study because many inflammatory markers were relatively low in normal-weight women and comparable to levels in normal-weight men. Unexpectedly, osteopontin and complement factor C5 were lower in normal-weight women than in normal-weight men. The regulation of osteopontin is incompletely understood. It is known to be synthesized by many different cell types and in response to different mediators (e.g., sex steroids and proinflammatory cytokines), which suggests that the lower concentrations in normal-weight women may be related to female sex hormones. Complement factors are predominantly produced in the liver, regulated by acute-phase response proteins or, in general, upon stimulation with cytokines. Complement factors, including C5, have been found to be lower in women than men in a previous study of individuals with normal weight; however, knowledge of the influence of sex on this biomarker is limited [27]. Moreover, we observed that vascular and endothelial activation marker sVCAM-1 was lower in normal-weight women than in normal-weight men, which is consistent with a lower CVD risk in women. sVCAM-1 mediates the attachment of immune cells to the endothelial cell layer, and chronically increased sVCAM-1 expression contributes to the development of atherosclerosis. It is unclear why sVCAM-1 differs between normal-weight men and women. It might be that the net effect of the many cytokines that jointly induce VCAM-1 expression is greater in men compared to women, while each individual cytokine is not changed significantly. At the vascular level, these cytokines may jointly activate endothelial cells to a greater extent in men resulting in higher VCAM-1 expression, shedding and release in the circulation [28,29].

Sex differences in circulating biomarkers were also observed in the obese condition. For instance, LPS-binding protein, MIF, and E-selectin were lower in obese women, while TNF-α was higher. LPS-binding protein is an acute-phase protein, and its expression increases in response to LPS, a bacterial component that is positively associated with gut permeability [30], as well as specific cytokine inducers (e.g., IL-1β and IL-6) [31]. Indeed, these cytokines were found to be higher in obese men. Furthermore, an amplifier of inflammatory signalling cascade, MIF, was increased in obese men. MIF is ubiquitously expressed, found in adipose tissue and immune cells (e.g., macrophages, T cells). Its expression has been related to adipocyte size [32] and severity of obesity-associated insulin resistance development [33]. E-selectin is also expressed by endothelial cells and similar to VCAM-1, is also involved in the recruitment of immune cells. The higher TNF-α levels in obese women might be related to sex-dependent differences in fat distribution and storage, as TNF-α is also predominantly expressed by subcutaneous tissue, the dominant form of fat storage in women [34]. Obese men tend to have more visceral fat than obese women, which is associated with a higher cardiometabolic risk, but we lack data on body composition, as mentioned in the limitations. Visceral fat is more prone to becoming inflamed than subcutaneous depots, and may secrete more pro-inflammatory factors [35], such as the cytokine MIF. Moreover, TIMP1, which is associated with increased risk of liver fibrosis [36], was found to be lower in women compared to men, and a similar sex difference was found for PAI-1. The exact reason why these biomarkers show sex differences is unclear.

Muscle health biomarkers were included in our study, since sex differences in muscle health are becoming apparent [37]. For example, the deterioration of muscle health such as frailty is more prevalent in females compared to males [38,39], whereas sarcopenia is more prevalent in males [40]. Interestingly, myostatin levels have been reported to be a potential biomarker for sarcopenia, which seems to be specific for males [40]. In our dataset, muscle-associated biomarkers myostatin, cathepsin B, galectin-1, irisin, and titin did not differ between sexes. Based on these findings and the aforementioned studies, we conclude that sex mainly plays a significant role in the deterioration of muscle health during the development of sarcopenia and should be taken into account in biomarker research in the context of sarcopenia and ageing.

Overall, our extensive analysis of biomarkers resulted in a large dataset that can help other researchers make informed choices on, for instance, the type of biomarkers to be analyzed and by which method (assay, kits, dilution), and how biomarkers are linked to organs or inflammatory and metabolic processes. This may be especially useful for rapid validation of results obtained from non-quantitative next-generation proteomics approaches (e.g., OLINK^®^). The small sample sizes are a limitation (e.g., risk of false positives), and we recommend that future studies on sex differences should be performed with larger cohorts and age-matched groups of females and males, with in-depth physiological phenotyping (e.g., sex hormone measurements, menstruation cycle, possible comorbidities and medication, checks for undiagnosed chronic diseases, leukocyte and platelet counts, etcetera). Such analyses would have supported mechanistic explanations provided here, which are based on literature and not on measurements (e.g., fat distribution, hormonal effects, metabolic predispositions). Future studies may better use blood collected in the fasted state, because this would, for instance, enable an analysis of fasting glucose levels and fasting triglyceride and cholesterol levels, which could provide an indication of an undiagnosed metabolic disorder.

We used ELISAs and electrochemiluminescence-based multiplex immunoassays from companies that provided us with sufficient information on how the assay was developed and validated, along with information of its use in published studies (e.g., the V-plex product line of MesoScale Discovery, Rockville, MD, USA). All assays employed herein were carefully tested and validated, and the analyses were performed in a standardized manner (same procedures and same plasma samples) to yield a comprehensive biomarker dataset with concentrations in normal-weight and obese individuals that may be used as guidance and reference for other researchers who wish to perform their own biomarker analyses or establish their own assays. Nevertheless, one constraint of the current dataset lies in the utilization of non-fasted plasma. Despite this limitation, the use of non-fasted plasma is common practice in various biomedical research fields, including socio-psychology, psychobiology, stress, and mental health [41,42,43].

The validation of the immunoassays used herein included the testing of (a) retrieval of a spiked antigen, (b) linear range of the calibration curve and (c) consistence in results of the same sample measured at different dilutions. Notably, although we have confirmed the analytical approach, assays and the values of many of the reported biomarkers in other cohort studies in healthy and metabolically compromised individuals [2,3,4,37,41,42,43,44,45,46], we do advise researchers to perform pilot analyses with the described assays because there can be subtle lot-to-lot variations in the assays themselves and differences in, for example, age or disease state, may require additional fine-tuning to establish an optimal dilution of plasma samples. Based on our experience with the described assays, the plasma biomarker concentrations provided herein can serve as an indication to save time and resources in future studies, thereby accelerating assay optimization.

## 4. Conclusions

We provided a comprehensive overview of biomarkers to gain insight into metabolic or inflammatory derangements related to obesity. Many biomarkers linked to chronic inflammation and dysmetabolism were elevated in persons with obesity, including several adipokines, interleukins, chemokines, acute-phase proteins, complement factors, and oxidized LDL. Further exploration suggests sex disparities in biomarker levels within both normal-weight and obese groups. Altogether, the methods and results provided in this study may help researchers to make informed choices regarding biomarkers analysis and assays and enable them to better design, analyze, and interpret their own studies, taking into account possible sex differences.

## 5. Materials and Methods

### 5.1. Blood Samples of Normal-Weight and Obese Women and Men

Biomarkers of metabolism and inflammation were measured in unfasted EDTA plasma samples. These plasma samples were from human blood donors and purchased from TCS Bioscience Ltd. (Buckingham, UK), a commercial provider of donor blood. More specifically, TCS Bioscience Ltd. collected the samples at the Plasma Donor Center Heidelberg (Heidelberg, Germany) from blood donors that provided consent to use the blood for research purposes, and according to the rules and ethical regulations set forward by the German Law on Blood Transfusions (“Transfusionsgesetz” with SOP 210-004). TCS Bioscience Ltd., anonymized the plasma samples prior to sending them to our laboratory. Anonymized plasma samples were obtained on 18 July 2018 (date of access for research purposes at TNO, Leiden, The Netherlands). No information that could identify individual participants was made available by TCS Biosciences Ltd. during or after data collection. Before plasma was accepted for donation, the donors underwent a medical check to make sure their health is adequate for donating plasma excluding diagnosed chronic disease. For this purpose, blood samples were also examined for diseases that are subject to mandatory reporting, such as HIV, hepatitis, and sexually transmitted diseases. Individuals who were suffering from a chronic disease or cancer, underwent major surgery, or had severe accidents in the past were unsuitable as donors and excluded. Blood samples were collected from both women and men with BMI < 25 (referred to as ‘normal-weight group’) and BMI > 30 (referred to as ‘obese group’). The age was comparable between all groups (no significant differences), and BMI was comparable in both sexes, i.e., both between males and females of the normal-weight group and the obese group (see details in Table 2). Most of the obtained plasma was used for setting up and optimizing assays prior to the actual analytical measurements presented herein. Because the amount of plasma was a limiting factor, the final analysis of biomarkers was conducted in randomly chosen *n* = 20 normal-weight and *n* = 20 obese persons. Consequently, sex was not optimally distributed resulting in variations in males and women per biomarker.

### 5.2. Biomarker Assays

The optimal dilution of EDTA plasma samples for each of the biomarkers was defined in pilot experiments using pooled plasma from normal-weight and obese individuals. Assays were performed according to protocols provided by the manufacturer and following an established optimization method to minimize sample volumes, as previously described [3,4]. Optimization of the different assays included the determination of the limit of quantification and the linear range, as well as the establishment of the optimal dilution within this linear range. Details about the providers of the respective assays and performance criteria of the ELISAs and electrochemiluminescence-based multiplex assays are provided in Supplementary Table S1. The ELISA assays listed therein were measured on a Biotek Synergy HTX machine (BioSPX, Abcoude, The Netherlands). The multiplex immunoassays were either MesoScale Discovery panels (Rockville, MD, USA) measured on a MESO QuickPlex SQ 120MM, or Quanterix panels (Billerica, MA, USA) measured on a SP-X Quanterix machine. Albumin, total protein, LDH, uric acid, creatine kinase, and urea nitrogen were analyzed using a Seamaty SMT-120VP machine (GBS, Leiden, The Netherlands). Globulin was calculated by the subtraction of total protein minus albumin. After defining optimal dilutions, the actual biomarker analyses were conducted, and we tested whether the plasma concentration of a biomarker differed between the obese and the normal-weight group, and exploratively studied sex differences by comparing obese women to obese men and normal-weight women to normal-weight men.

### 5.3. Statistics

We tested the null hypothesis, that the plasma concentration of a particular biomarker did not differ in concentration, in three different comparisons: (1) obese subjects vs. normal-weight subjects, (2) obese women vs. obese men, and (3) normal-weight women vs. normal-weight men. Due to the exploratory nature of our research, uncorrected *p*-values are reported. Statistical analysis was performed using IBM SPSS statistics version 25.0 (SPSS Inc., Chicago, IL, USA). Data was tested for normality with the Shapiro–Wilk test and for equal variance with Levene’s test (α = 0.05). For normally distributed variables, an independent sample *t*-test was used (2-sided). In case the data was not normally distributed, a Mann–Whitney U test was used (2-sided). *p*-values < 0.05 were considered statistically significant. The results of these analyses are displayed using a volcano plot and a correlation plot. For the latter, Pearson regression analysis was performed to compare the change in the expression of biomarker from the normal-weight condition to the obese condition (in women and men) and calculate the correlation coefficient.

## Figures and Tables

**Table 1:** Circulating biomarkers concentrations in obese and normal-weight women and man.

	Normal-Weight Men	Normal-Weight Women	Mean Normal Weight	Obese Men	Obese Women	Mean Obese
**Adipokines**						
Adiponectin [µg/mL]	3.74 ± 1.17	4.80 ± 1.46	4.27 ± 1.40	2.49 ± 0.82	3.38 ± 1.22	2.85 ± 1.07 **#**
Angiopoietin-like 4 [µg/mL]	1.02 ± 1.83	0.34 ± 0.25	0.68 ± 1.32	0.33 ± 0.14	0.42 ± 0.53	0.36 ± 0.34
Leptin [ng/mL]	7.91 ± 2.50	12.20 ± 9.88 *	10.97 ± 8.55	22.88 ± 18.96	68.74 ± 46.52 *	41.23 ± 39.20 **#**
Resistin [ng/mL]	16.61 ± 8.40	17.18 ± 4.23	16.90 ± 6.48	18.82 ± 8.29	18.62 ± 3.85	18.74 ± 6.72
Visfatin [ng/mL]	11.12 ± 6.02	9.63 ± 6.34	10.37 ± 6.06	7.36 ± 5.89	6.54 ± 3.53	7.03 ± 4.98
**Onset and amplification of inflammation**						
Calprotectin S100A8 [ng/mL]	34.24 ± 47.84	18.77 ± 19.73	24.19 ± 32.03	31.99 ± 32.64	16.74 ± 20.61	26.66 ± 29.37
Calprotectin S100A8/S100A9 [µg/mL]	0.86 ± 1.01	0.56 ± 0.47	0.66 ± 0.70	1.04 ± 0.85	0.60 ± 0.55	0.88 ± 0.77
CD14 [µg/mL]	1.40 ± 0.20	1.55 ± 0.31	1.50 ± 0.28	1.33 ± 0.19	1.35 ± 0.15	1.34 ± 0.17 **#**
IFN-g [pg/mL]	0.15 ± 0.18	0.14 ± 0.21	0.15 ± 0.19	0.09 ± 0.06	0.16 ± 0.09	0.12 ± 0.08
IL-1β [pg/mL]	0.37 ± 0.22	0.27 ± 0.20	0.32 ± 0.21	0.45 ± 0.39	0.65 ± 0.46	0.53 ± 0.42
IL-1Ra [ng/mL]	0.82 ± 1.55	0.49 ± 1.22	0.65 ± 1.36	0.61 ± 0.54	0.78 ± 0.75	0.68 ± 0.62
IL-6 [pg/mL]	0.89 ± 0.47	0.97 ± 0.39	0.93 ± 0.42	1.60 ± 0.97	2.39 ± 1.21	1.92 ± 1.11 **#**
IL-8 [pg/mL]	9.05 ± 4.58	7.41 ± 4.12	8.23 ± 4.33	8.00 ± 4.41	9.45 ± 8.84	8.58 ± 6.37
IL-10 [pg/mL]	1.05 ± 1.02	0.39 ± 0.15	0.72 ± 0.79	0.59 ± 0.18	0.59 ± 0.25	0.59 ± 0.20
IL-13 [pg/mL]	1.54 ± 1.55	1.28 ± 1.55	1.41 ± 1.51	1.14 ± 0.92	0.68 ± 0.75	0.95 ± 0.87
IL-18 [ng/mL]	0.52 ± 0.31	0.39 ± 0.12	0.45 ± 0.24	0.74 ± 0.39	0.51 ± 0.27	0.65 ± 0.35 **#**
MIF [ng/mL]	2.39 ± 2.95	1.09 ± 1.42	1.55 ± 2.10	1.72 ± 0.72	0.97 ± 0.52 *	1.46 ± 0.74
MPO [ng/mL]	43.47 ± 14.66	35.61 ± 27.88	39.54 ± 22.05	42.10 ± 20.90	38.17 ± 10.21	40.53 ± 17.18
TGF-β [ng/mL]	27.41 ± 6.44	24.67 ± 8.72	26.04 ± 7.59	25.04 ± 11.64	21.22 ± 7.49	23.51 ± 10.14
TNF-α [pg/mL]	3.11 ± 1.70	2.57 ± 1.23	2.84 ± 1.47	2.54 ± 0.53	5.05 ± 3.60 *	3.54 ± 2.55
TNF-RII [ng/mL]	2.89 ± 0.73	3.53 ± 0.96	3.21 ± 0.90	2.58 ± 0.86	2.80 ± 0.50	2.67 ± 0.73 **#**
CCL2/MCP-1 [pg/mL]	120.16 ± 27.31	87.16 ± 18.78 *	98,71 ± 26.82	128.90 ± 22.29	91.59 ± 24.81 *	115.84 ± 29.01
CCL3/MIP-1a [pg/mL]	10.64 ± 4.87	12.65 ± 4.41	11.94 ± 4.55	17.34 ± 3.63	38.43 ± 46.18	24.72 ± 28.02
CCL5 [ng/mL]	74.79 ± 23.40	65.47 ± 31.46	70.13 ± 27.41	88.84 ± 70.07	72.72 ± 102.42	82.39 ± 82.30
CXCL1/GROa [pg/mL]	153.69 ± 64.34	189.70 ± 121.93	177.10 ± 104.92	383.88 ± 356.74	246.05 ± 111.53	335.64 ± 298.08 **#**
Fractalkine [ng/mL]	6.82 ± 1.74	7.56 ± 1.52	7.31 ± 1.60	6.15 ± 0.98	6.20 ± 1.19	6.17 ±1.03 **#**
CXCL4/PF-4 [µg/mL]	5.34 ± 3.05	4.48 ± 1.88	4.91 ± 2.51	4.61 ± 2.35	3.80 ± 1.81	4.28 ± 2.14
CXCL7/NAP-2 [µg/mL]	2.77 ± 1.09	2.25 ± 0.76	2.51 ± 0.95	2.70 ± 1.21	2.14 ± 0.77	2.47 ± 1.07
CXCL10/IP10 [pg/mL]	278.15 ± 192.44	260.32 ± 80.94	266.56 ± 126.13	361.36 ± 144.09	319.68 ± 74.20	346.77 ± 123.56
CXCL11/I-TAC [pg/mL]	42.40 ± 17.37	61.75 ± 23.90	54.98 ± 23.36	60.33 ± 24.11	66.93 ± 28.91	62.64 ± 25.33
CXCL12/SDF-1a [pg/mL]	1652.16 ± 403.53	1899.55 ± 347.81	1812.96 ± 377.47	1518.75 ± 663.21	1265.63 ± 363.44	1430.16 ± 578.66 **#**
Osteopontin [ng/mL]	40.33 ± 16.13	25.49 ± 14.53 *	32.91 ± 16.77	31.84 ± 11.82	26.36 ± 8.81	29.65 ± 10.82
**Complement system**						
C1q [µg/mL]	26.62 ± 3.85	29.63 ± 3.34	28.12 ± 3.84	34.34 ± 5.16	33.89 ± 5.72	34.16 ± 5.24 **#**
C3 [mg/mL]	0.56 ± 0.06	0.53 ± 0.08	0.54 ± 0.07	0.65 ± 0.13	0.66 ± 0.10	0.66 ± 0.12 **#**
C5 [µg/mL]	72.61 ± 4.80	81.51 ± 7.78 *	77.06 ± 7.77	85.54 ± 12.73	90.25 ± 18.29	87.42 ± 14.92 **#**
**Acute-phase response**						
hs-CRP [µg/mL]	0.73 ± 0.47	0.84 ± 0.84	0.80 ± 0.72	1.81 ± 1.40	2.30 ± 1.95	2.02 ± 1.62 **#**
Haptoglobin [mg/mL]	0.89 ± 0.70	0.55 ± 0.35	0.67 ± 0.51	1.03 ± 0.49	1.21 ± 0.36	1.09 ± 0.45 **#**
SAA [µg/mL]	2.35 ± 0.90	2.46 ± 1.27	2.41 ± 2.15	5.31 ± 3.88	5.48 ± 5.59	5.38 ± 4.50 **#**
LPS-binding protein [µg/mL]	3.65 ± 1.29	3.87 ± 1.03	3.76 ± 1.14	4.00 ± 1.19	2.95 ± 0.79 *	3.58 ± 1.15
α1-antichymotrypsin [µg/mL]	157.50 ± 44.72	193.24 ± 73.22	175.37 ± 61.83	209.72 ± 81.66	174.08 ± 25.21	195.46 ± 66.45
Fetuin-A [mg/mL]	0.65 ± 0.11	0.74 ± 0.27	0.69 ± 0.20	0.73 ± 0.13	0.75 ± 0.31	0.74 ± 0.21
Fetuin-B [µg/mL]	3.92 ± 0.52	3.96 ± 1.05	3.94 ± 0.80	4.17 ± 0.53	4.20 ± 0.77	4.18 ± 0.62
**Oxidative stress**						
Oxidized LDL [U/L]	40.23 ± 9.00	41.84 ± 14.64	41.27 ± 12.71	54.17 ± 16.75	52.30 ± 14.67	52.52 ± 15.68 **#**
**Liver health**						
Albumin [g/L]	44.81 ± 2.64	41.95 ± 4.96	42.95 ± 4.59	44.53 ± 2.03	43.07 ± 1.75	44.02 ± 2.02
Globulin [g/L]	22.61 ± 3.35	20.35 ± 5.08	21.14 ± 4.59	26.12 ± 3.40	28.71 ± 2.75	27.03 ± 3.36 **#**
ALT [U/L]	18.18 ± 17.52	11.73 ± 4.59	13.99 ± 10.97	28.78 ± 12.06	24.70 ± 20.56	27.35 ± 15.14 **#**
AST [U/L]	23.23 ± 5.62	23.91 ± 5.81	23.67 ± 5.61	29.44 ± 11.74	29.00 ± 15.29	29.29 ± 12.69
**Fibrosis**						
Galectin-3 [ng/mL]	6.18 ± 2.94	3.97 ± 2.31	5.07 ± 2.81	5.31 ± 2.76	4.48 ± 2.29	4.98 ± 2.55
Hyaluronic acid [ng/mL]	33.01 ± 19.52	24.52 ± 10.67	28.77 ± 15.92	78.89 ± 119.27	40.93 ± 16.30	63.71 ± 93.26 **#**
TIMP-1 [ng/mL]	135.43 ± 16.37	122.91 ± 26.45	129.17 ± 22.35	151.57 ± 33.67	108.34 ± 14.20 *	134.28 ± 34.68
**Iron metabolism**						
TfR [µg/mL]	1.16 ± 0.14	1.31 ± 0.29	1.26 ± 0.26	1.50 ± 0.25	1.40 ± 0.30	1.46 ± 0.26 **#**
Transferrin (mg/mL)	4.56 ± 1.10	4.78 ± 1.22	4.70 ± 1.15	4.00 ± 0.58	4.07 ± 0.69	4.03 ± 0.60 **#**
Hepcidin [ng/mL]	6.62 ± 7.36	6.98 ± 10.20	6.86 ± 9.10	10.30 ± 7.26	14.54 ± 18.30	11.78 ± 11.97
EPO [mIU/mL]	107.55 ± 265.27	46.70 ± 117.93	67.99 ± 178.58	9.69 ± 10.10	8.14 ± 11.38	9.15 ± 10.29
**Vascular and endothelial activation**						
sICAM-1 [ng/mL]	219.18 ± 43.11	207.70 ± 41.01	213.44 ± 41.37	239.95 ± 50.34	220.41 ± 40.39	232.14 ± 46.52
E-selectin [ng/mL]	6.02 ± 2.60	6.43 ± 1.72	6.24 ± 2.12	7.60 ± 2.72	5.02 ± 1.44 *	6.57 ± 2.59
*p*-selectin [ng/mL]	79.98 ± 20.38	73.67 ± 26.47	76.82 ± 23.22	81.24 ± 32.42	65.71 ± 19.58	75.02 ± 28.47
sVCAM-1 [ng/mL]	487.98 ± 86.00	395.22 ± 68.86 *	441.60 ± 89.52	418.92 ± 75.02	437.28 ± 170.26	426.26 ± 118.42
**Coagulation: thrombosis and fibrinolysis**						
Alpha-1 antitrypsin [mg/mL]	2.72 ± 0.52	3.02 ± 1.13	2.92 ± 0.95	2.77 ± 0.50	2.95 ± 0.67	2.84 ± 0.55
Fibrinogen [mg/mL]	2.68 ± 0.86	2.73 ± 0.94	2.70 ± 0.88	3.02 ± 1.41	3.02 ± 0.92	3.02 ± 1.21
PAI-1 [ng/mL]	34.55 ± 12.21	27.33 ± 9.62	30.94 ± 11.32	49.54 ± 19.65	25.24 ± 14.33 *	39.82 ± 21.18
vWF-A2 [ng/mL]	1.39 ± 1.35	1.04 ± 0.56	1.21 ± 1.03	1.02 ± 0.39	0.92 ± 0.34	0.98 ± 0.36
ADAMTS13 [ng/mL]	323.95 ± 187.45	260.14 ± 108.36	290.37 ± 150.20	185.26 ± 86.58	116.42 ± 80.53	175.43 ± 86.41 **#**
ANXA3 [ng/mL]	3.87 ± 1.75	2.10 ± 1.00	2.72 ± 1.54	2.44 ± 1.04	1.91 ± 0.81	2.25 ± 0.98
**Blood pressure modulators**						
Angiotensinogen [ng/mL]	465.16 ± 530.28	215.21 ± 201.32	400.44 ± 464.80	365.59 ± 444.63	117.98 ± 84.44	181.18 ± 173.53
ACE [ng/mL]	146.94 ± 118.06	113.39 ± 69.41	130.17 ± 95.82	129.77 ± 58.35	131.16 ± 77.15	130.36 ± 64.86
**Neurological factors**						
BDNF [ng/mL]	13.98 ± 4.16	14.84 ± 4.77	14.41 ± 4.38	14.04 ± 5.89	11.15 ± 3.70	12.88 ± 5.22
NF-light [pg/mL]	9.21 ± 4.33	7.17 ± 1.76	7.88 ± 2.98	8.60 ± 4.70	9.25 ± 7.63	8.83 ± 5.69
IGF2 [ng/mL]	59.49 ± 9.96	69.22 ± 14.24	64.35 ± 12.96	59.14 ± 15.53	61.91 ± 12.25	60.25 ± 14.03
IGFBP7 [ng/mL]	143.60 ± 15.76	167.26 ± 101.46	155.43 ± 71.70	153.49 ± 29.18	154.51 ± 27.59	153.90 ± 27.82
S100B [pg/mL]	108.61 ± 50.63	143.08 ± 88.34	131.01 ± 77.61	116.82 ± 81.56	100.16 ± 50.41	110.99 ± 71.21
**Muscle health**						
Myostatin (pg/mL)	519.29 ± 887.59	89.45 ± 150.28	239.89 ± 554.34	132.96 ± 75.89	81.42 ± 189.04	114.92 ± 124.73
Cathepsin B [ng/mL]	39.58 ± 5.36	38.67 ± 12.47	38.99 ± 10.37	38.71 ± 13.96	35.70 ± 15.25	37.66 ± 14.10
Galectin-1 [ng/mL]	40.76 ± 23.95	25.68 ± 10.02	30.39 ± 16.49	32.55 ± 8.12	34.08 ± 18.03	33.08 ± 12.04
Creatine kinase [U/L]	158.71 ± 70.18	99.31 ± 48.59 *	120.10 ± 62.38	206.15 ± 124.64	232.57 ± 216.90	215.40 ± 157.60 **#**
Irisin [pg/mL]	2296.07 ± 647.72	2060.82 ± 681.41	2135.1 ± 662.3	2575.71 ± 890.23	2331.79 ± 1106.02	2490.34 ± 949.25
Titin [pmol/L]	104.42 ± 100.91	77.21 ± 62.26	86.74 ± 76.42	211.03 ± 195.96	270.70 ± 379.75	231.91 ± 265.79 **#**
**Kidney health**						
Urea nitrogen [mmol/L]	5.04 ± 1.46	3.65 ± 1.06 *	4.14 ± 1.36	5.05 ± 1.0	3.71 ± 0.58 *	4.58 ± 1.14
**Others**						
LDH [U/L]	223.71 ± 42.89	209.23 ± 37.86	214.30 ± 39.19	364.54 ± 150.24	276.71 ± 60.15	333.80 ± 131.32 **#**
Uric acid [umol/L]	279.87 ± 41.89	203.93 ± 48.66 *	230.51 ± 58.57	324.98 ± 87.57	246.57 ± 87.97	297.53 ± 93.59 **#**
Cystatin C [µg/mL]	1.07 ± 0.16	1.31 ± 0.68	1.19 ± 0.49	1.18 ± 0.21	1.05 ± 0.20	1.13 ± 0.21
THBS4 [ng/mL]	393.94 ± 100.91	368.22 ± 111.26	377.22 ± 105.79	606.08 ± 231.67	578.28 ± 202.54	596.35 ± 216.88 **#**
Meteorin-like [ng/mL]	1.95 ± 0.42	1.66 ± 0.20	1.80 ± 0.36	1.47 ± 0.33	1.53 ± 0.18	1.49 ± 0.27 **#**
PAM [ug/mL]	0.74 ± 0.07	0.73 ± 0.12	0.73 ± 0.11	0.67 ± 0.07	0.66 ± 0.06	0.67 ± 0.07

Data are presented as mean ± SD. The name of the biomarker is indicated in the first column. Several statistical comparisons were made. Significant differences between obese and normal-weight subjects independent of sex are indicated by the pound (**#**) symbol and shown in blue. Significant differences in normal-weight women compared to normal-weight men are indicated by an asterisk (*) and shown in orange. Similarly, significant differences between obese women and obese men are indicated by an asterisk (*) and shown in orange. Abbreviations (in alphabetical order): ACE, angiotensin converting enzyme; ADAMTS13, a disintegrin and metalloproteinase with thrombospondin notifs 13; ALT, alanine aminotransferase; ANGTPL4 = Angiopoietin like-4; ANXA3 = annexin A3; AST, aspartate aminotransferase; BDNF, brain-derived neurotrophic factor; CCL5, C-C motif chemokine ligand 5 (also referred to as RANTES); CK = creatine kinase; CRP, C-reactive protein; CD14, cluster of differentiation 14; CXCL, C-X-C motif chemokine ligand; EPO, erythropoietin; FGF21, fibroblast growth factor 21; Hp = haptoglobin; sICAM-1, soluble intracellular cell adhesion molecule-1; IFN-g, interferon gamma; IL, interleukin (various); IL-1Ra, interleukin-1 receptor antagonist; IGF2, insulin growth factor 2; IGFBP7, insulin growth factor binding protein 7; LDH, Lactate dehydrogenase; LGALS1 = galectin 1; LPS-BP, LPS binding protein; LPTN = leptin; MCP-1, monocyte chemoattractant protein-1; MIF, macrophage migration inhibitory factor; MIP-1alpha = macrophage inflammatory protein-1 alpha (or CCL3); MPO, myeloperoxidase; MSTN = myostatin; NF-light, neurofilament light chain; oxLDL, oxidized low density lipoprotein; PAI-1, plasminogen activator inhibitor-1; PAM = peptidyl glycine alpha-amidating monooxygenase; S100B, S100 calcium-binding protein B; SAA, serum amyloid A; SDF-1a = stromal cell-derived factor 1 alpha; TfR, transferrin receptor; TIMP-1, tissue inhibitor of metalloproteinases-1; sVCAM-1, soluble vascular cell adhesion molecule-1; TfR = transferrin receptor; TGF-beta, transforming growth factor-beta; THSB4 = thrombospondin-4; TNF-a, tumour necrosis factor-alpha; TNFRII, tumour necrosis factor receptor II; vWF, van Willebrand factor.

**Table 2:** Age and BMI of blood donors.

	Normal Weight Men (*n* = 17)	Normal Weight Women	Mean Normal Weight (*n* = 40)	Obese Men (*n* = 25)	Obese Women (*n* = 15)	Mean Obese (*n* = 40)
Age	28.9 ± 12.5	30.4 ± 12.1	29.8 ± 12.1	37.3 ± 12.9	31.5 ± 10.1	35.1 ± 12.1
BMI	22.3 ± 2.1	22.1 ± 1.6	22.2 ± 1.8	34.8 ± 3.1	36.7 ±6.1	35.5 ± 4.5 #

Data are presented as mean ± SD. Significant differences between obese and normal-weight subjects (independent of sex) are indicated with the pound (#) symbol and the colour blue.

## Data Availability

Data are available upon reasonable request.

## Supplementary Materials

The following supporting information can be downloaded at https://www.mdpi.com/article/10.3390/ijms26157576/s1.
