# Supplementary material for: Biomarkers of Metabolism and Inflammation in Individuals with Obesity and Normal Weight: A Comparative Analysis Exploring Sex Differences
Source: Int J Mol Sci. 2025 Aug 5;26(15):7576. doi: 10.3390/ijms26157576 (PMC12347797; doi:10.3390/ijms26157576)
Supplement: Supplementary file 1 [file ijms-26-07576-s001.zip › ijms-3754758-supplementary.pdf]

Supplemental Table 1: Biomarkers and details of respective assays in alphabetical order

Biomarkers	Catalog Nr.	Company	Linear range	Limit of quantification	Dilution factor used in this study
ACE [ng/ml]	DY929	R&D Systems	125 to 8000 pg/mL	125 pg/mL	200x
ADAMTS13 (ng/ml)	DY4245	R&D Systems	0.78125 to 50 ng/mL	0.78125 ng/mL	65x
Adiponectin [µg/ml]	DY1065	R&D Systems	62.5 to 4000 pg/mL	62.5 pg/mL	15000x
Albumin [g/L]	AW01980	Seamaty, (Hamburg, Germany)	10-70 g/L	10 g/L	2x
Alpha 2-macroglobulin (mg/ml)	DY1938	R&D Systems	0.15625 to 10 ng/mL	0.15625 ng/mL	5,8E5x
ALT [U/l]	10745138 202	Roche Diagnostics (Rotkreuz, Switzerland)	5 to 2000 U/L	5 U/L	1x
Angiopoietin-like 4/ Fasting induced adipose factor [µg/ml]	DY3485	R&D Systems	1.25 to 80 ng/mL	1.25 ng/mL	22x
Angiotensinogen [ng/ml]	DY3156	R&D Systems	187.5 to 12000 pg/mL	187.5 pg/mL	180x
ApoB100	RAB0610	Sigma	2.4576 to 600 ng/mL	2.4576 ng/mL	100000x
AST [U/l]	10745120 202	Roche Diagnostics	5 to 500 U/L	5 U/L	1x
BDNF [ng/ml]	DY248	R&D Systems	23.438 to 1500 pg/mL	23.438 pg/mL	50x
C1q [µg/ml]	BMS2099	Invitrogen/ Thermo Fisher Scientific (Waltham USA)	0.9375 to 60 ng/mL	0.9375 ng/mL	1300x
C3 [mg/ml]	NBP2-60618	Novus Biologicals	0.46875 to 30 µg/mL	0.46875 µg/mL	300x
C5 [µg/ml]	NBP2-60620	Novus Biologicals	0.156 to 10 ng/mL	0.156 ng/mL	60000x
Calprotectin S100A8 [ng/ml]	DY4570	R&D Systems	31.25 to 2000 pg/mL	31.25 pg/mL	80x
Calprotectin S100A8/S100A9 [µg/ml]	DY8226	R&D Systems	93.75 to 6000 pg/mL	93.75 pg/mL	800x
Cathepsin B [ng/ml]	DY2176	R&D Systems	62.5 to 4000 pg/mL	62.5 pg/mL	100x
CCL5 [ng/ml]	DY278	R&D Systems	15.625 to 1000 pg/mL	15.625 pg/mL	750x
CD14 [µg/ml]	DY383	R&D Systems	62.5 to 4000 pg/mL	62.5 pg/mL	3300x

Creatine kinase [U/L]	AW01980	Seamaty	25-1000U/L	25 U/L	2x
hs-CRP [µg/ml]	DY1707	R&D Systems (Abingdon, UK)	15.6 to 1000 pg/mL	15.6 pg/mL	10000x
CXCL4/PF-4 [µg/ml]	DY795	R&D Systems	15.625 to 1000 pg/mL	15.625 pg/mL	10000x
CXCL7/NAP-2 [µg/ml]	DY393	R&D Systems	15.625 to 1000 pg/mL	15.625 pg/mL	19000x
CXCL10/IP-10 [pg/ml]	DIP100	R&D Systems	7.813 to 500 ng/mL	7.813 ng/mL	2x
CXCL1 – GROa	K15067M -1	Meso Scale Diagnostics	2.91-11900 pg/mL	2.91 pg/mL	2x
Cystatin C [µg/ml]	DY1196	R&D Systems	62.5 to 2000 pg/mL	62.5 pg/mL	3600x
EPO [mIU/ml]	RAB0654-1KT	Sigma-Aldrich (Amsterdam, Netherlands)	0.137 to 100 mIU/mL	0.137 mIU/mL	4x
E-selectin [ng/ml]	DY724	R&D Systems	93.75 to 6000 pg/mL	93.75 pg/mL	16x
Fetuin-A [mg/ml]	DFTA00	R&D Systems	7.8 to 500 ng/mL	7.8 pg/mL	10000x
Fetuin-B [µg/ml]	DY1725	R&D Systems	93.75 to 6000 pg/mL	93.75 pg/mL	4500x
FGF21 [pg/ml]	DY2539	R&D Systems	31.25 to 2000 pg/mL	31.25 pg/mL	1x
Fibrinogen [mg/ml]	NBP2-60465	Novus Biologicals (Wiesbaden, Germany)	0.16 to 40 µg/mL	0.16 µg/mL	1000x
Galectin-3 [ng/ml]	DY1154	R&D Systems	62.5 to 4000 pg/mL	62.5 pg/mL	7x
Haptoglobin [mg/ml]	DY8465	R&D Systems	31.25 to 2000 pg/mL	31.25 pg/mL	2000000x
Hepcidin [ng/ml]	DY8307	R&D Systems	3.125 to 200 pg/mL	3.125 pg/mL	5x-450x
Hyaluronic acid [ng/ml]	DY3614	R&D Systems	0.37 to 90 ng/mL	0.37 ng/mL	5x
ICAM-1 [ng/ml]	DY720	R&D Systems	31.25 to 2000 pg/mL	31.25 pg/mL	750x
IFN- γ [pg/ml]	85-0329	Quanterix (Billerica, USA)	0.012 to 50 pg/mL	0.012 pg/mL	4x
IL-1b [pg/ml]	85-0329	Quanterix	0.024 to 100 pg/mL	0.098 pg/mL	4x
IL-1Ra [ng/ml]	DY280	R&D Systems	39.1-2500 pg/mL	39.1 pg/mL	1x
IL-6 [pg/ml]	85-0329	Quanterix	0.073 to 300 pg/mL	0.073 pg/mL	4x
IL-8 [pg/ml]	85-0329	Quanterix	0.098 to 400 pg/mL	0.392 pg/mL	4x
IL-10 [pg/ml]	85-0329	Quanterix	0.024 to 100 pg/mL	0.024 pg/mL	4x

IL-13 [pg/ml]	K15049D	MesoScale Diagnostics (Rockville, USA)	0.24 to 353 pg/mL	0.24 pg/mL	2x
IL-18 [ng/ml]	DY318	R&D Systems	11.719 to 750 pg/mL	11.719 pg/mL	5x
IGF2 (ng/ml)	CSB-E04583h	Cusabio	62.5 to 4000 pg/mL	62.5 pg/mL	1800x
IGFB7 (pg/ml)	DY1334	R&D Systems	39.0625 to 2500 pg/ml	39.0625 pg/mL	580x
Insulin [mU/l]	10-1132-01	Mercodia (Uppsala, Sweden)	0.15 to 20 mU/L	0.15 mU/L	1x-40x
IP-10 (CXCL10)	K15067M-1	Meso Scale Diagnostics	13.8-56500 pg/mL	13.8 pg/mL	2x
Irisin [pg/ml]	DY9420	R&D Systems	250 to 4000 pg/mL	250 pg/ml	3x
I-TAC (CXCL11)	K15067M-1	Meso Scale Diagnostics	1.83-7500 pg/mL	7.32 pg/mL	2x
Lactate [nmol/µl]	MAK064-1KT	Sigma-Aldrich	0.04 to 0.2 nmol/µL	0.04 nmol/µL	50x
LDH [U/L]	AW01980	Seamaty	20-1500 U/L	20 U/L	2x
Leptin [ng/ml]	DY398	R&D Systems	31.25 to 2000 pg/mL	31.25 pg/mL	100x
LPS-binding protein [µg/ml]	DY870	R&D Systems	0.78125 to 50 ng/mL	0.78125 ng/mL	600x
Meteorin-like [ng/ml]	DY7867	R&D Systems	15.625 to 1000 pg/mL	15.625 pg/mL	12x
MCP-1	K15067M-1	Meso Scale Diagnostics	7.73-31650 pg/mL	7.73 pg/mL	2x
MIF [ng/ml]	DY289	R&D Systems	31.25 to 2000 pg/mL	31.25 pg/mL	10x
MIP-1a	K15067M-1	Meso Scale Diagnostics	7.02-28750 pg/mL	28.1 pg/mL	2x
MPO [ng/ml]	DY3174	R&D Systems	62.5 to 4000 pg/mL	62.5 pg/mL	120x
Myostatin (pg/ml)	DY788	R&D Systems	31.25 to 2000 pg/mL	31.25 pg/mL	2x-10x
NF-light [pg/ml]	20-8002	Uman	0.2 to 40 pg/mL	0.2 pg/mL	6x
Osteopontin [ng/ml]	DY1433	R&D Systems	62.5 to 4000 pg/mL	62.5 pg/mL	60x
Oxidized LDL [U/l]	10-1143-01	Mercodia	1.57 to 22.2 mU/L	1.57 mU/L	9200x
PAI-1 [ng/ml]	DY9387	R&D Systems	7.8125 to 500 pg/mL	7.8125 pg/mL	600x
P-selectin [ng/ml]	DY137	R&D Systems	125 to 8000 pg/mL	125 pg/mL	80x
Resistin [ng/ml]	DY1359	R&D Systems	31.25 to 2000 pg/mL	31.25 pg/mL	60x

S100B [pg/ml]	DY4570	R&D Systems	31.25 to 2000 pg/mL	31.25 pg/mL	2x
SAA [µg/ml]	DY3019	R&D Systems	1.5625 to 100 ng/mL	1.5625 ng/mL	180x
SDF-1a	K15067M-1	Meso Scale Diagnostics	118-484500 pg/mL	473 pg/mL	2x
SerpinA3/α1-antichymotrypsin [µg/ml]	NBP-60541	Novus Biologicals	1.125 to 72 ng/mL	1.125 ng/mL	24000x
TfR [µg/ml]	DY2474	R&D Systems	78.125 to 5000 pg/mL	78.125 pg/mL	2000x
TGF-β [ng/ml]	DY240	R&D Systems	31.25 to 2000 pg/mL	31.25 pg/mL	100x
THBS4 [ng/ml]	EH473RB	Invitrogen/Thermo Fisher Scientific	0.102 to 25 ng/mL	0.102 ng/mL	260x
TIMP-1 [ng/ml]	DY970	R&D Systems	31.25 to 2000 pg/mL	31.25 pg/mL	500x
Titin [pmol/L]	27902	Immuno-Biological Lab. (Gunma, Japan)	46.88 to 1500 pmol/L	46.88 pmol/L	3x
TNF-α [pg/ml]	85-0329	Quanterix	0.098 to 400 pg/mL	0.098 pg/mL	4x
TNF-RII [ng/ml]	DY726	R&D Systems	7.813 to 500 pg/mL	7.813 pg/mL	45x
Total protein [g/L]	AW01980	Seamaty	15-110 g/L	15 g/L	2x
Transferrin [mg/ml]	80736	Chrystal Chem	9.375 to 600 ng/mL	9.375 ng/mL	65000x
TREM-2 (pg/ml)	DY1828	R&D Systems	23.4375 to 1500 pg/mL	23.4375 pg/mL	4x
Urea nitrogen [umol/L]	AW01980	Seamaty	0.9-35.7 umol/L	0.9 umol/L	2x
Uric acid [umol/L]	AW01980	Seamaty	50-1100 umol/L	50 umol/L	2x
VCAM-1 [ng/ml]	DY809	R&D Systems	15.625 to 1000 pg/mL	15.625 pg/mL	3000x
Visfatin [ng/ml]	NBP2-76710	Novus Biologicals	0.313 to 20 ng/mL	0.313 ng/mL	7.5x
vWF-A2 [ng/ml]	DY2764	R&D Systems	46.875 to 3000 pg/mL	46.875 pg/mL	3.5x

Abbreviations: ACE, angiotensin converting enzyme; ADAMTS13, A Disintegrin And Metalloproteinase with Thrombospondin Motifs 13; ALT, alanine aminotransferase; ANGTPL4=Angiopoietin like-4; ANXA3= annexin A3; AST, aspartate aminotransferase; ApoB, apolipoprotein B; BDNF, brain-derived neurotrophic factor; CCL5, C-C motif chemokine ligand 5 (also referred to as RANTES); CK= creatine kinase; CRP, C-reactive protein; CD14, cluster of differentiation 14; CXCL, C-X-C motif chemokine ligand; EPO, erythropoietin; FGF21, fibroblast growth factor 21; Hp = haptoglobin; sICAM-1, soluble intracellular cell adhesion molecule-1; IFN-gamma, interferon-gamma; IL, interleukin (various); IL-1Ra, interleukin-1 receptor antagonist; IGF2, insulin growth factor 2; IGFBP7, insulin growth factor binding protein 7; IP-10, Interferon gamma-induced protein 10; I-TAC, Interferon-inducible T-cell alpha chemoattractan; LDH, Lactate dehydrogenase; LGALS1 = Galectin 1; LPS-BP, LPS binding protein; LPTN = Leptin; MCP-1, monocyte chemoattractant protein-1; MIF, macrophage migration inhibitory factor; MIP-1alpha= Macrophage Inflammatory Protein-1 alpha (or CCL3); MPO, myeloperoxidase; MSTN=Myostatin; NF-light, neurofilament light chain; oxLDL, oxidized low density lipoprotein; PAI-1, plasminogen activator inhibitor-1; PAM= Peptidylglycine Alpha-Amidating Monooxygenase; S100B, S100

calcium-binding protein B; SAA, serum amyloid A; SDF-1a= stromal cell-derived factor 1 alpha; TfR, transferrin receptor; TIMP-1, tissue inhibitor of metalloproteinases-1; sVCAM-1, soluble vascular cell adhesion molecule-1; TfR= transferrin receptor; TGF-beta, transforming growth factor-beta; THSB4= thrombospondin-4; TNF-alpha, tumor necrosis factor-alpha; TNFRII, tumor necrosis factor receptor II; TREM2, Triggering receptor expressed on myeloid cells-2; vWF, van Willebrand factor.
